# Medical Jurisprudence

**Published:** 1892-05

**Authors:** Henry A. Riley

**Affiliations:** New York


### Progress in Medical Science.

### MEDICAL JURISPRUDENCE.

CONDUCTED BY

HENRY A. RILEY, A. B., LL. B., New York.

### LIFE INSURANCE AND DEATH CERTIFICATES.

Physicians, in the course of their professional duty, are necessarily required to furnish certificates of the causes of death for various purposes. This is required of them by the health laws in most cities, and the certificates are of great value in making up the vital statistics, upon which much of our sanitary and health legislation is based. For failure to file such a certificate in the proper office is generally a misdemeanor, and punishable by fine and imprisonment, or both.

It is often the case also that, in claims under life insurance policies, one of the most important "proofs of death" is the certificate of the attending physician, giving the cause of death, with other information.

Does the physician violate any law of professional ethics in giving such a certificate, and does he break the statute law, which, in most States, provides that the physician is not allowed to disclose any information obtained by him in a professional capacity, and which is necessary for the proper treatment of the patient? These are important and interesting questions.

In the case of the reports to the health boards, there can be no question that the physician is obliged to make such reports, and that no rule of professional etiquette is violated by doing so.

In the other case of certificates to insurance companies, a somewhat different question is involved. Both of these points were raised, however, in a recent New York case, and the decision will be of interest.

The cause of death, in this instance, was delirium tremens, and an admission of it would prevent a recovery on the part of an infant beneficiary who could not protect himself.

The attending physician made a report, both to the insurance company and the Board of Health, and gave the cause of death as delirium tremens. Did he wrong the beneficiary by so doing? On the trial, the company offered both certificates in evidence as conclusive evidence against the plaintiff. The Court held, however, that they could not be admitted so as to bind the infant. In the case of the Board of Health report, it was held that public records of this description were not competent evidence in actions between private parties where the cause of death was the main question in issue. It must be proved in other ways.

In regard to the report to the insurance company, the Court held that it would have been a conclusive admission against the plaintiff if he were an adult, but that the rights of an infant ward could not be lost by the admission of his guardian, and the certificate was, on this ground, refused as evidence.

The policy of insurance did not require the cause of death to be stated, but only called for satisfactory proof of death, and this could be furnished without disclosing the cause. The act of the physician in furnishing the certificate was not considered as reprehensible, because he was asked to give it by the guardian of the infant, and it was supposed to be necessary to secure payment, although, in fact, it was the most efficient means of preventing a recovery.

It would be well for physicians to remember that, in cases of suicide, etc., a statement of the cause of death may be prejudicial and may also be entirely unnecessary.

---

### THE PROPER TREATMENT OF CRIMINALS.

An article in the New York *Independent*, not long since, gave a careful *résumé* of the Penal Code of Italy, which went into operation January 1, 1890. The writer states that the only sound basis of penal punishment is protection of public order and private rights. He repudiates, as unphilosophical and untrue, the theory that the infliction of punishment is because the offender has done wrong. This elimination of the moral element in crime is rather startling, and the writer says that his theory has not been put in practice by the Italian Code, and laments that the best opportunity of "making a memorable epoch in the progress of scientific legislation" has been lost.

The following paragraph will be of interest, as stating clearly the writer's position:

But, in nearly all cases, there is better use for a man than to bury him in prison. It is this conviction that has dictated the Penal Code of New Zealand, under which none but immediately dangerous convicts are imprisoned, all others being placed under the guardianship and tutelage of such reputable citizens as will undertake the charge.

This system has built up a public spirit in the community, such that men of wealth, especially of large estates in land, vie with one another in assuming the care of youthful offenders, and rescuing them from a criminal career.

It has been, in part, copied in Massachusetts, where the system of probation officers and parole, practised so successfully of late years for juvenile offenders, is now extended to all who are convicted for the first time of less serious crimes.

There is reason to hope that it will profoundly modify the penal codes of all civilized nations, since the limited experience of it hitherto indicates that it is the surest and most efficient means ever devised of preventing the formation and growth of a criminal class.

---

### A NEW YORK MALPRACTICE CASE.

A MALPRACTICE suit has just been tried against a physician of Fishkill Landing, New York, with the result of awarding a verdict of $2,500 damages against him. Some time since a drayman fell from his cart and broke his arm. The bone protruded from the flesh, and the wound was filled with dirt. It was dressed and looked after by the physician, but the patient found it necessary to enter St. Luke's Hospital, New York, after about a month, at which time the arm was swollen to double its usual size. When he left the hospital, the arm was useless and will always remain so. Suit was therefore brought for malpractice, the claim being made that the wound was not properly cleansed. The damages were laid at $5,000 and a verdict given for $2,500.

---

### MUSIC AND CHURCH BELLS IN COURT.

THE ringing of church bells has often proved to be a nuisance, by reason of the long continuance or untimeliness of the clanging, and the aid of the law has been invoked frequently to prohibit it. This has been the case in England and also in several of the states of our own country. In a case reported not long since in the newspapers, a curious question, not of bells but of music, arose. The chorister of a church was discharged for some reason, and took his revenge by sitting in the congregation and singing purposely out of tune. The congregation apparently did not know what to do, and an application for an injunction was thought of, but, we believe, not tried. An arrest for disturbing public worship would probably have met the exigencies of the occasion. This last case occurred in North Carolina, which seems to be particularly affected by musical troubles.

In one instance, occurring not long since, a female prisoner was solacing the tedium of her enforced retirement by singing, although the wife of the jailor was sick, and he requested her to stop. When she refused to do so, the jailor severely beat her with a horsewhip. This was considered a harsher form of criticism than the rendering of the music demanded, and upon a trial the jailor was sentenced to pay a fine of $100. In another case, also in North Carolina, not music but public worship was involved, as it seems that the defendant engaged in a prize fight near a church during service. Some one happened to announce the fact in church, whereupon the congregation ran out. The accused, rather curiously, was acquitted of the charge of disturbing public worship.

